# Identification and validation of oxidative stress and immune-related hub genes in Alzheimer’s disease through bioinformatics analysis

**DOI:** 10.1038/s41598-023-27977-7

**Published:** 2023-01-12

**Authors:** Shengjie Li, Jinting Xiao, Chuanjiang Huang, Jikui Sun

**Affiliations:** 1 Department of Neurosurgery, The First Affiliated Hospital of Shandong First Medical University & Shandong Provincial Qianfoshan Hospital, Jinan, 250000 China; 2 Department of Neurosurgery, Jiangxi Provincial People’s Hospital, The First Affiliated Hospital of Nanchang Medical College, Nanchang, 330000 China; 3 Nanchang University, Nanchang, 330000 China; 4 Department of Medical Ultrasound, The First Affiliated Hospital of Shandong First Medical University & Shandong Provincial Qianfoshan Hospital, Jinan, 250000 China

**Keywords:** Biomarkers, Health care, Neurology

## Abstract

Alzheimer’s disease (AD) is the leading cause of dementia in aged population. Oxidative stress and neuroinflammation play important roles in the pathogenesis of AD. Investigation of hub genes for the development of potential therapeutic targets and candidate biomarkers is warranted. The differentially expressed genes (DEGs) in AD were screened in GSE48350 dataset. The differentially expressed oxidative stress genes (DEOSGs) were analyzed by intersection of DEGs and oxidative stress-related genes. The immune-related DEOSGs and hub genes were identified by weighted gene co-expression network analysis (WGCNA) and protein–protein interaction (PPI) analysis, respectively. Enrichment analysis was performed by Gene Ontology and Kyoto Encyclopedia of Genes and Genomes. The diagnostic value of hub genes was assessed by receiver operating characteristic analysis and validated in GSE1297. The mRNA expression of diagnostic genes was determined by qRT-PCR analysis. Finally, we constructed the drug, transcription factors (TFs), and microRNA network of the diagnostic genes. A total of 1160 DEGs (259 up-regulated and 901 down-regulated) were screened in GSE48350. Among them 111 DEOSGs were identified in AD. Thereafter, we identified significant difference of infiltrated immune cells (effector memory CD8 T cell, activated B cell, memory B cell, natural killer cell, CD56 bright natural killer cell, natural killer T cell, plasmacytoid dendritic cell, and neutrophil) between AD and control samples. 27 gene modules were obtained through WGCNA and turquoise module was the most relevant module. We obtained 66 immune-related DEOSGs by intersecting turquoise module with the DEOSGs and identified 15 hub genes through PPI analysis. Among them, 9 hub genes (CCK, CNR1, GAD1, GAP43, NEFL, NPY, PENK, SST, and TAC1) were identified with good diagnostic values and verified in GSE1297. qRT-PCR analysis revealed the downregulation of SST, NPY, GAP43, CCK, and PENK and upregulation of NEFL in AD. Finally, we identified 76 therapeutic agents, 152 miRNAs targets, and 91 TFs regulatory networks. Our study identified 9 key genes associated with oxidative stress and immune reaction in AD pathogenesis. The findings may help to provide promising candidate biomarkers and therapeutic targets for AD.

## Introduction

Alzheimer’s disease (AD) is an insidious, progressive, and devastating neurodegenerative disease, which is recognized as the leading cause of dementia in aged population. As a global health challenge, AD affects about 47 million people worldwide, with an estimated number of cases increasing to 152 million by 2050^1,2^. Pathologic features of AD are characterized by persistent deposition of the intercellular amyloid β-peptide (Aβ) plaques and intracellular tau protein and impairment of the neuron-to-neuron synaptic communication and nutrient transportation inside neuron^3^. Despite recent advances in new understanding of AD pathogenesis and improved management strategies, the mechanism underlying AD pathogenesis is not completely understood. Importantly, so far, there is no effective intervention strategy in preventing or curing of AD. The reported death rate of AD increased more than 145% between 2000 and 2019^4^. There is an impendency to further explore the underlying mechanism of AD initiation and progression for developing effective intervention strategies.

Oxidative stress and neuroinflammation have been implicated in the pathogenesis of AD and the acceleration of neurodegeneration^5,6^. Oxidative stress is involved in the modulation of gene expression patterns and metabolic activities, characterized by the disruption of redox homeostasis^7^. Previous in vitro and in vivo studies revealed that oxidative stress caused by the accumulation of Aβ contributed to the initiation of AD^8^. Aβ-mediated oxidative stress results in mitochondrial dysfunction, impairment of glucose metabolism, loss of proteostasis and synaptic plasticity, altered signal transduction, neuroinflammation, and progressive loss of neurons^9^. Neuroinflammation has also been identified as another crucial component of AD pathogenesis. Glial cell activation is found in early AD, even before Aβ accumulation^10,11^. Upon AD-related proteins stimulation, activated microglia cells accumulate around amyloid plaques, responsible for the activation of innate immune response and maintenance of oxidative microenvironment. Activated microglia cells eventually lead to exacerbation of neuronal degeneration and death through producing cytokines, chemokines, reactive oxygen species, and nitric oxide^12^. Besides, microglia-mediated neuroinflammation is also involved in the genetics and neuropathology of late-onset AD^13^. Apart from microglia, astrocyte, an important player in maintenance of homoeostasis of brain tissue microenvironment, is found not to be an innocent bystander in AD pathogenesis^14^. In addition to internalization and degradation of Aβ, activated astrocyte is likely involved in exacerbating neuroinflammation through releasing cytokine, interleukin, nitric oxide, and other potentially cytotoxic molecules^15^. Emerging evidence has suggested that the disease severity, individual differences, and complicated pathogenesis of AD may be attributed to multiple genes and their variants^15–17^. Therefore, identification and comprehensive analysis of potential candidate genes will deepen our understanding of the gene regulation in oxidative stress and immune reaction, which may provide promising candidate biomarkers and therapeutic targets for AD.

## Materials and methods

### Data sources

**Figure 1:**
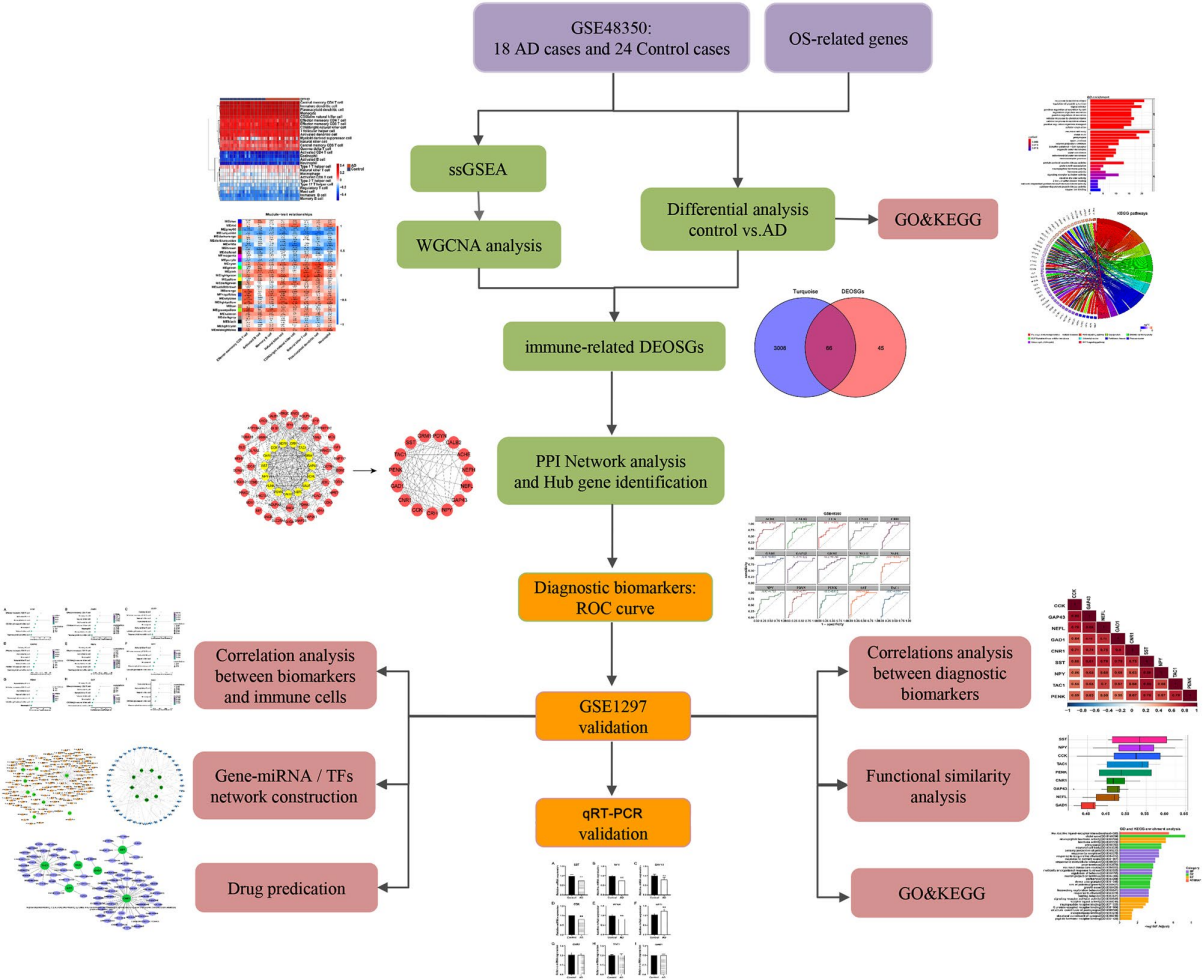
Workflow to identify oxidative stress and immune-related genes of Alzheimer’s disease. *WGCNA* weighted gene co-expression network analysis, *GO* gene ontology, *KEGG* kyoto encyclopedia of genes and genomes, *DEOSGs* differentially expressed oxidative stress genes, *PPI* protein–protein interaction, *TFs* transcription factors, *qRT-PCR* quantitative real-time PCR, *ROC* receiver operating characteristic.

All the data we used in our study are publicly accessible at NCBI GEO (Accession Number: GSE48350 and GSE1297) database (https://www.ncbi.nlm.nih.gov/geo/). The GSE48350 dataset, contained 253 samples, was based on GPL570 platform (HG-U133_Plus_2). Sample inclusion criteria were (1) hippocampal tissue; (2) age over 60-year-old. Because all genes expression levels in GSM300182 (Control) and GSM1176215 (AD) were the same, these two samples were considered abnormal and were eliminated. Data of 24 hippocampal control and 18 hippocampal AD samples were extracted for analysis. GSE1297 was based on GPL96 [HG-U133A] Affymetrix Human Genome U133A Array, which included hippocampal samples of 22 AD patients and 9 normal people. The inclusion criteria of GSE1297 were the same as those of GSE48350. GSE48350 was used as the training set and GSE1297 was used as the external validation set. 1399 oxidative stress-related genes (OSGs) were obtained from the GeneCards (https://www.genecards.org/)^18^. The workflow of this study was shown in Fig. 1.

### Identification of differentially expressed genes (DEGs)

The Limma package was applied to identify the DEGs in AD^19^. DEGs were generated according to the following criterion: adj. P < 0.05. The results of DEGs were visualized by the heatmap and volcano plot, which were made by using “pheatmap” and “ggplot2” R packages. The differentially expressed oxidative stress genes (DEOSGs) were screened by the intersection of DEGs and OSGs with “VennDiagram” R package^20^.

### Functional enrichment analysis

The clusterProfiler package was applied to perform the Gene Ontology (GO) and Kyoto Encyclopedia of Genes and Genomes (KEGG) enrichment analysis. GO analysis encompassed cellular component, biological function, and molecular function. A P < 0.05 was considered statistically significant. KEGG was performed to analyze the associated enrichment pathways. Adjust P-value < 0.05 was considered statistically significant.

### Immune infiltration analysis

Gene Set Variation Analysis (GSVA) is a non-parametric, unsupervised algorithm. GSVA R package was applied in ssGSEA for the analysis of immune cell infiltration in AD and control samples^21^, which including activated CD4 T cell, activated B cell, activated CD8 T cell, activated dendritic cell, CD56bright natural killer cell, CD56dim natural killer cell, central memory CD4 T cell, effector memeory CD4 T cell, central memory CD8 T cell, effector memeory CD8 T cell, gamma delta T cell, macrophage, eosinophil, immature B cell, immature dendritic cell, mast cell, MDSC, memory B cell, monocyte, neutrophil, plasmacytoid dendritic cell, regulatory T cell, T follicular helper cell, natural killer cell, natural killer T cell, Type 1 T helper cell, Type 2 T helper cell, and Type 17 T helper cell. Metagene of 28 immune cell subtypes was obtained from https://www.cell.com/cms/10.1016/j.celrep.2016.12.019/attachment/f353dac9-4bf5-4a52-bb9a-775e74d5e968/mmc3.xlsx^22^. We compared the difference in proportion of immune cells in AD and control samples via Wilcoxon tests. A P*-*value ≤ 0.05 was considered statistically significant*.*

### Weighted gene co-expression network analysis (WGCNA)

WGCNA is a systematic biological method used to describe gene association patterns between different samples. We performed WGCNA by using R package^23^. To detect outliers and remove abnormal samples, we established clustering tree map. WGCNA was carried out according to the gene expression profiles extracted from GSE48350 dataset and data collected from previous analysis of immune infiltration in AD and control samples. The best soft-thresholding power β was chosen according to the criteria of approximate scale-free topology. The adjacency matrix was transformed to a topological overlap matrix for the construction of network analysis. The gene dendrogram and module color were generated based on the degree of dissimilarity. Subsequently, the initial modules were further divided according to the dynamic tree cut and merged with similar modules. After that, the correlations between modules and differentially infiltrating immune cells were calculated. The module which was most closely associated with the differentially infiltrating immune cells, was identified for subsequent analysis. The intersection of DEOSGs and genes in key modules were carried out by using the “VennDiagram” R package. The shared genes were defined as immune-related DEOSGs, which were used for subsequent analysis.

### Protein–protein interaction (PPI) network construction

The immune-related DEOSGs were uploaded to the Search Tools for the Retrieval of Interacting Genes (STRING, http://www.string-db.org/)^24^. In the PPI network analysis, a confidence > 0.4 was defined as the cut-off criterion. MCODE plug-in (degree cutoff = 2, node score cutoff = 0.2, K-core = 2, and max depth = 100) in Cytoscape (https://cytoscape.org) was applied for the analysis of key gene modules in the PPI network. Key modules genes were defined as hub genes.

### Receiver operating characteristic (ROC) curve analysis

To verify the accuracy of screened hub genes, we performed ROC curve and area under the curve (AUC) analysis by using the “pROC” package. Genes with AUC > 0.7 were identified as useful for disease diagnosis. Two gene expression datasets including GSE48350 and GSE1297 were applied for expression pattern analysis of hub genes generated in previous section. Boxplots of gene expression profile of hub genes were generated by using “ggplot2” in R package.

### Quantitative real-time PCR (qRT-PCR) analysis of diagnostic genes based on clinical samples

We performed qRT-PCR analysis to verify the expression of diagnostic genes in peripheral blood of AD patients. The blood samples were obtained from 9 AD patients and 9 healthy control individuals. Briefly, total RNA from peripheral blood was extracted using Takara RNAiso Plus (9108) Trizol reagent. After assessment of RNA quality and concentration, reverse transcription and qRT-PCR were performed using the Takara PrimeScript RT Master Mix (RR036A) and SYBR Green Premix (RR420A), respectively. Primer sequences of target genes were as follows: forward 5′- CCCCAGACTCCGTCAGTTTCT-3′, reverse 5′-CATTCTCCGTCTGGTTGGGT-3′ for SST; forward 5′-TGTTCCCAGAACTCGGCTTG-3′, reverse 5′-TGCA TTGGTAGGA TGGGTGG-3′ for NPY; forward 5′-GAGCAGCCAAGCTGAAGAGAAC-3′, reverse 5’-GCCATTTCTTAGAGTTCAGGCATG-3’ for GAP43; forward 5′-CCAAGACCTCCTCAACGTGAAG-3′, reverse 5′-ATGCTTCCCACGCTGGTGAAAC-3′ for NEFL; forward 5′-TGAGGGTATCGCAGAGAACGGA-3′, reverse 5′-CGGTCACTTATCCTGTGGCTGG-3′ for CCK; forward 5′-CTGTTCCTCACAGCCATCGACA-3′, reverse 5′-TGGCTATGGTCCACATCAGGCA-3′ for CNR1; forward 5′-CTGAATTACTGGTCCGACTG-3′, reverse 5′-AGAACTGCTGAGGCTTGG-3′ for TAC1; forward 5′-TGGTTTTTAGGGGTTTTTTTTTTTGGA-3', reverse 5′-ACAAATACACCCCCTTTAATCTACTCTCC-3' for GAD1; forward 5′-TGCAGGTTTCCCAAATTTTC-3′, reverse 5′-GTGCAGCTACCGCCTAGTG-3′ for PENK. qRT-PCR was performed in technical triplicate for per target gene. Relative transcript abundance was determined by using the ΔΔCt method and normalized to the averaged mRNA expression levels of β-actin. The protocol was reviewed and approved by the Ethics Committee of the First Affiliated Hospital of Shandong First Medical University & Shandong Provincial Qianfoshan Hospital.

### Correlation analysis of diagnostic genes and functional similarity analysis of diagnostic genes

Corrplot package was applied for the correlation analysis of diagnostic gene. GOSemSim in R package (http://bioconductor.org/packages/2.6/bioc/html/GOSemSim.html) was used to perform GO semantic similarity analysis^25^. The potential diagnostic biomarker was evaluated by using the geometric mean of semantic similarities.

### Correlation analysis between infiltrating immune cells and diagnostic genes

We calculated the Spearman correlation coefficient between diagnostic genes and differentially infiltrating immune cells by using “psych” package^26^. The results were visualized by “ggpubr” package^27^. Spearman's correlation coefficients ranged between -1 and 1. The coefficients − 1, 0 and 1 indicated negative, no, and positive correlation, respectively.

### Drug predication for diagnostic genes

To screen candidate drugs targeting diagnostic genes, we utilized the Drug Gene Interaction Database (DGIdb) (https://dgidb.genome.wustl.edu)^28^. The results were visualized in Cytoscape.

### Construction of gene-miRNA regulatory network

To construct gene-miRNA regulatory network, we utilized the miRNet database (https://www.mirnet.ca/)^29^. Cytoscape was applied for the visualization of regulatory network. In the network, a green round dot represented a diagnostic gene and a light orange triangle dot represented a miRNA.

### Construction of gene-transcription factor regulatory network

To construct gene-transcription factor (TF) regulatory network, we exploited the Network Analyst database (https://www.networkanalyst.ca/)^30^. Cytoscape was applied for the visualization of regulatory network. In the network, a green round dot represented a diagnostic gene and a blue triangle dot represented a TF.

### Statistical analysis

All statistical analysis were performed by using R software (v.4.1.0). Wilxcon test was utilized to compare the differences in immune cells between two groups. Spearman correlation coefficients were calculated to determine the correlation of diagnostic genes. ROC curves were generated with the R package “pROC”, and the corresponding AUC values were calculated. Student’s *t* test was used to determine the differences of qRT-PCR data between two groups. A p-value or if necessary adjusted P-value < 0.05 was considered statistically significant.

## Results

### Identification of DEGs and DEOSGs in AD

**Figure 2:**
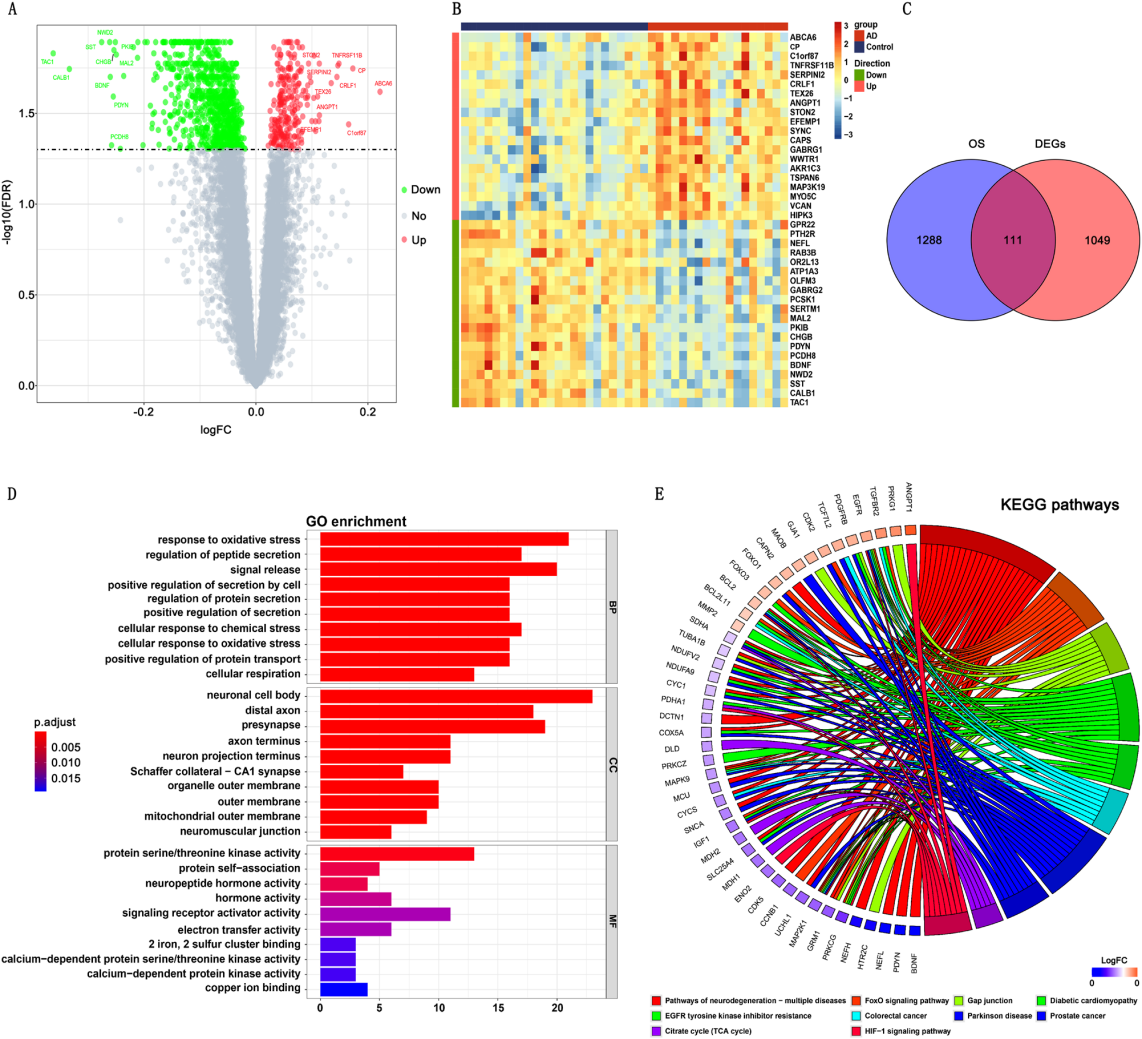
(**A**) Volcano plots of differentially expressed genes (DEGs). The red dots represent upregulated genes, and the green dots represent downregulated genes. (**B**) Heatmaps of DEGs. (**C**) An overlap of 111 DEOSGs between DEGs and OS-related genes in Venn diagram. (**D**) GO analysis of DEOSGs. (**E**) KEGG pathway enrichment analysis of DEOSGs.

1160 DEGs were identified from GSE48350 dataset, included 259 upregulated and 901 downregulated genes (AD vs. Control). Figure 2A,B demonstrated the volcano plots and heatmaps of DEGs. The Venn diagrams showed that 111 DEOSGs were overlapped between DEGs and OSGs (Fig. 2C). The GO analysis suggested that DEOSGs were mainly enriched in oxidative stress response, regulation of peptide secretion, neuronal cell body, distal axon, protein serine/threonine kinase activity, and protein self-association (Fig. 2D). The KEGG enrichment analysis revealed that the DEOSGs were predominately related to pathways involved in neurodegeneration-multiple diseases, FoxO signaling pathway, and gap junction (Fig. 2E).

### Immune infiltrating cell analysis of AD

**Figure 3:**
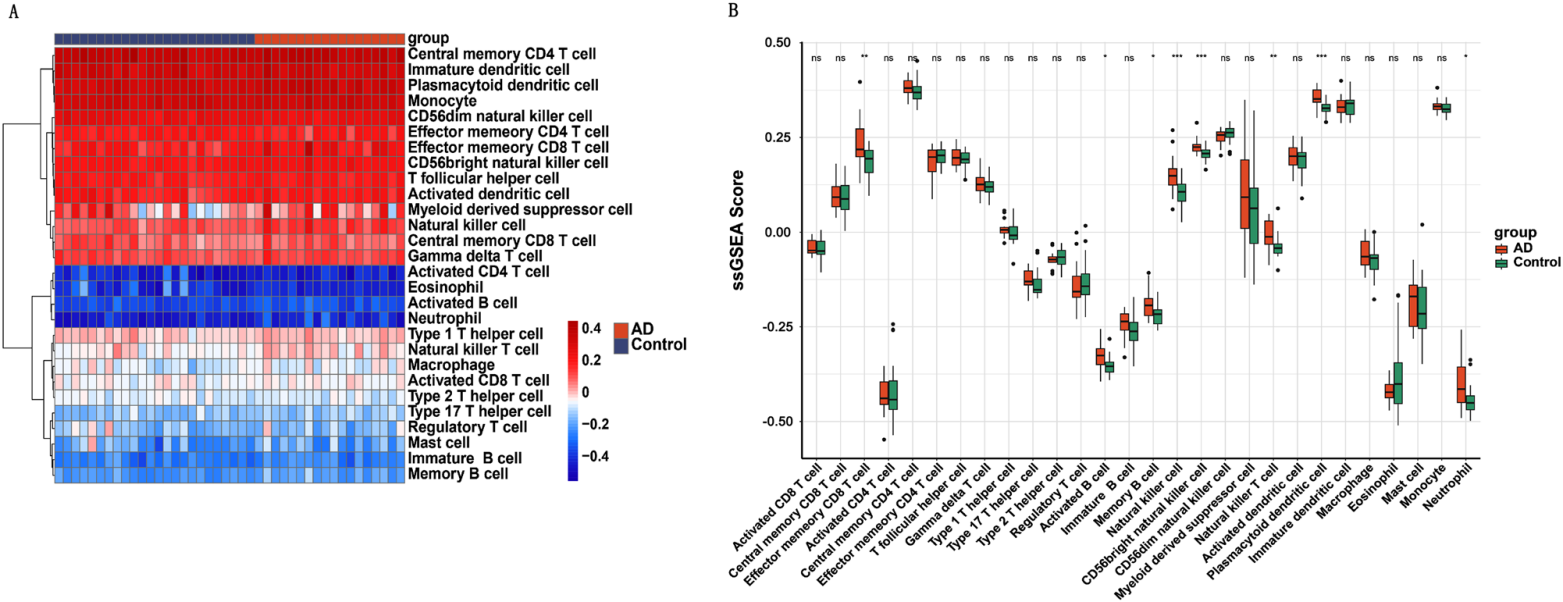
(**A**) Heatmap showing the distribution of 28 infiltrating immune cells in AD and normal samples. (**B**) ssGSEA analysis of immune infiltration of 28 immune cells.

The profile of immune infiltration in AD was explored by using ssGSEA. The distribution of 28 infiltrating immune cells was demonstrated in the heatmap (Fig. 3A). Abundance of effector memory CD8 T cell, activated B cell, memory B cell, natural killer cell, CD56 bright natural killer cell, natural killer T cell, plasmacytoid dendritic cell, and neutrophil were found significantly higher in AD samples compared to those in control group (Fig. 3B). This result indicated the critical role played by immune cells in the pathogenesis of AD.

### Identification of the key module and genes associated with oxidative stress and immune reaction in AD

**Figure 4:**
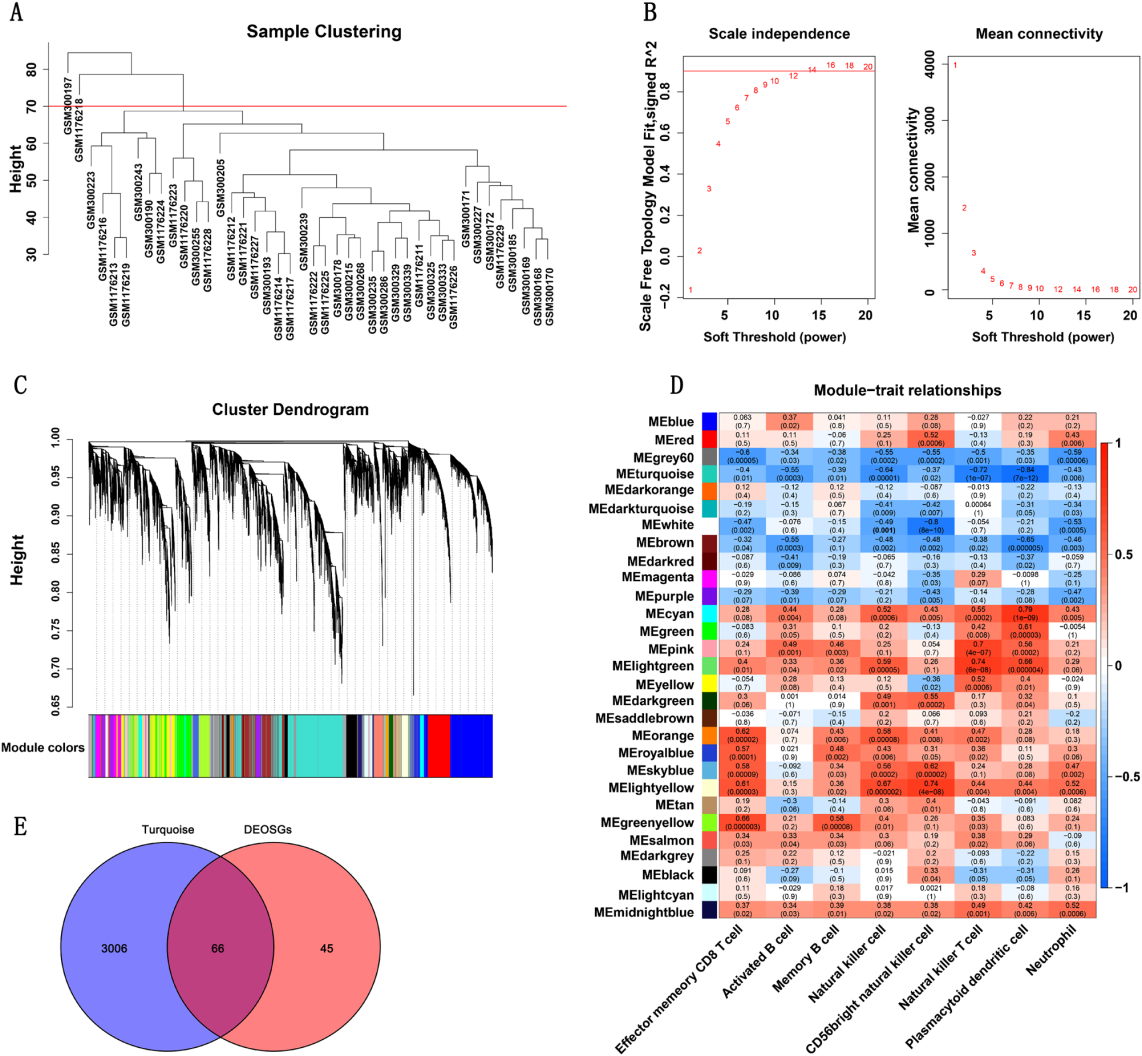
(**A**) A sample dendrogram showed two abnormal samples were removed. (**B**) Analysis of the scale-free fit index and mean connectivity through Scale-free network construction. (**C**) Clustering dendrogram. (**D**) Heatmap of module-trait relationships. The turquoise module was the most relevant module associated with plasmacytoid dendritic cell. (**E**) An overlap of 66 immune-related DEOSGs by intersection of DEOSGs and genes in turquoise modules.

A sample dendrogram showed that two abnormal samples were removed, and 40 samples were analyzed (Fig. 4A). The best soft-threshold power of 14 was selected based on the construction of scale-free network (Fig. 4B). 27 gene modules were obtained through the construction of co-expression matrix (Fig. 4C). Based on the module-trait relationships in Fig. 4D, we found that the turquoise module was the one with highest relevance to plasmacytoid dendritic cell (Cor = − 0.84, p = 7e − 12). Therefore, the turquoise module was used for downstream analysis. We took the intersection of DEOSGs and genes in turquoise modules and identified 66 immune-related DEOSGs (Fig. 4E).

### Identification of hub genes associated with oxidative stress and immune reaction in AD

**Figure 5:**
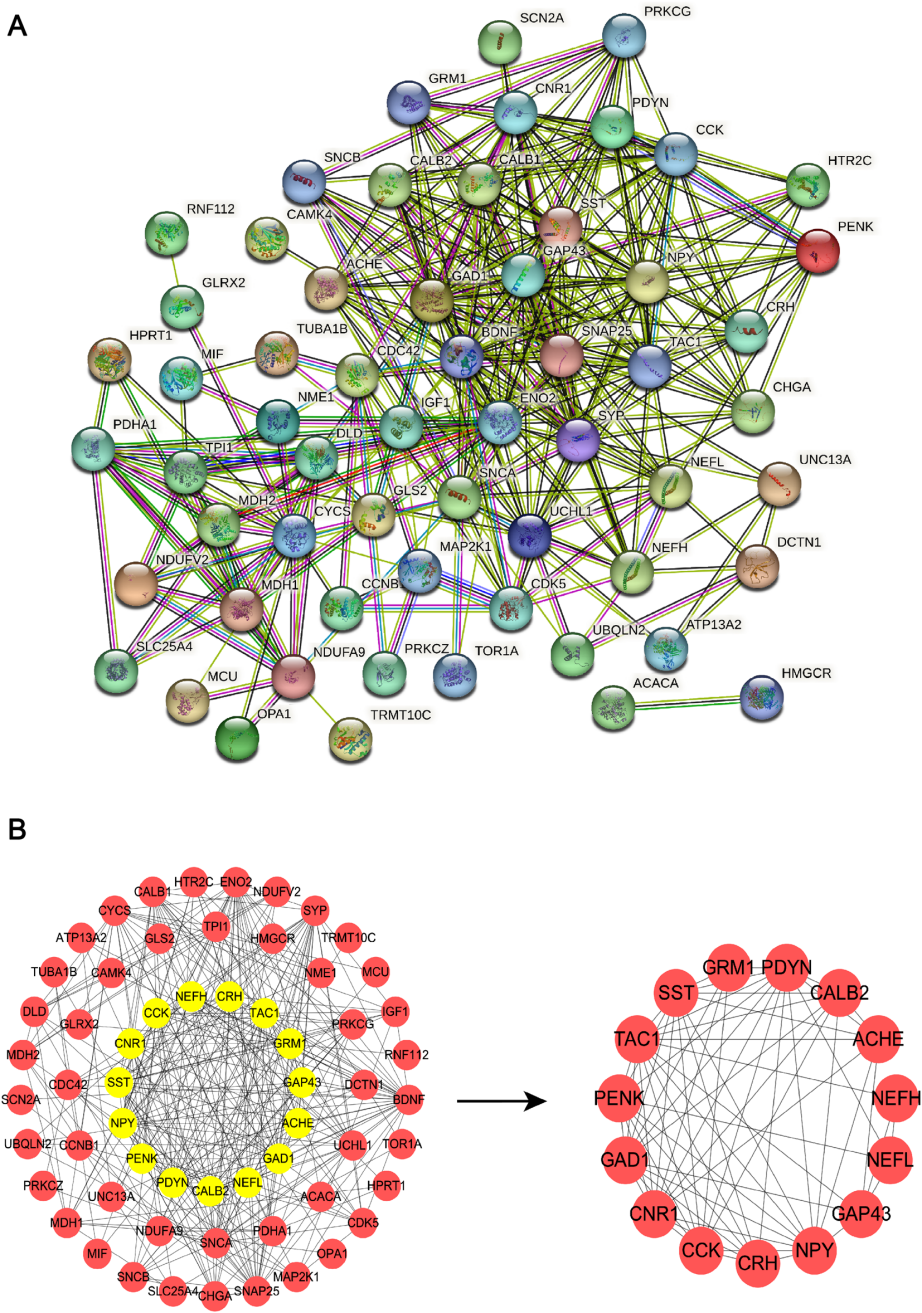
(**A**) PPI network construction for identification of hub genes. (**B**) 15 key modules genes (hub genes) were visualized by PPI network.

PPI network was displayed in Fig. 5A. 15 hub genes (CCK, ACHE, GRM1, GAD1, TAC1, PENK, NEFH, CALB2, NPY, CNR1, GAP43, CRH, PDYN, NEFL, and SST) in AD were identified by using MCODE plug-in Cytoscape, and the PPI network of 15 hub genes was shown in Fig. 5B.

### Evaluation of the diagnostic value of hub genes in AD

**Figure 6:**
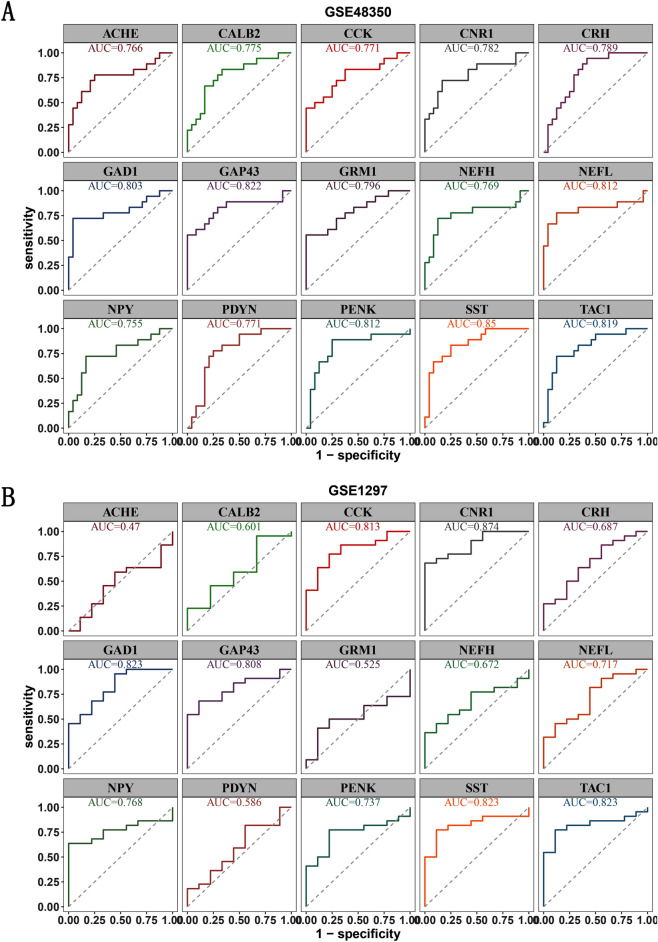
ROC curve validated the diagnostic significance of hub genes for AD in training dataset GSE48350 (**A**) and external validation dataset GSE1297 (**B**). The AUC areas of 9 hub genes (CCK, CNR1, GAD1, GAP43, NEFL, NPY, PENK, SST, and TAC1) were greater than 0.7 in two datasets.

We evaluated the diagnostic performance of hub genes by plotting ROC curves of GSE48350 and GSE1297 (Fig. 6A,B). The AUC values of 9 hub genes (CCK, CNR1, GAD1, GAP43, NEFL, NPY, PENK, SST, and TAC1) were larger than 0.7 in two datasets, which indicated that these hub genes possessed favorable diagnostic values in AD.

**Figure 7:**
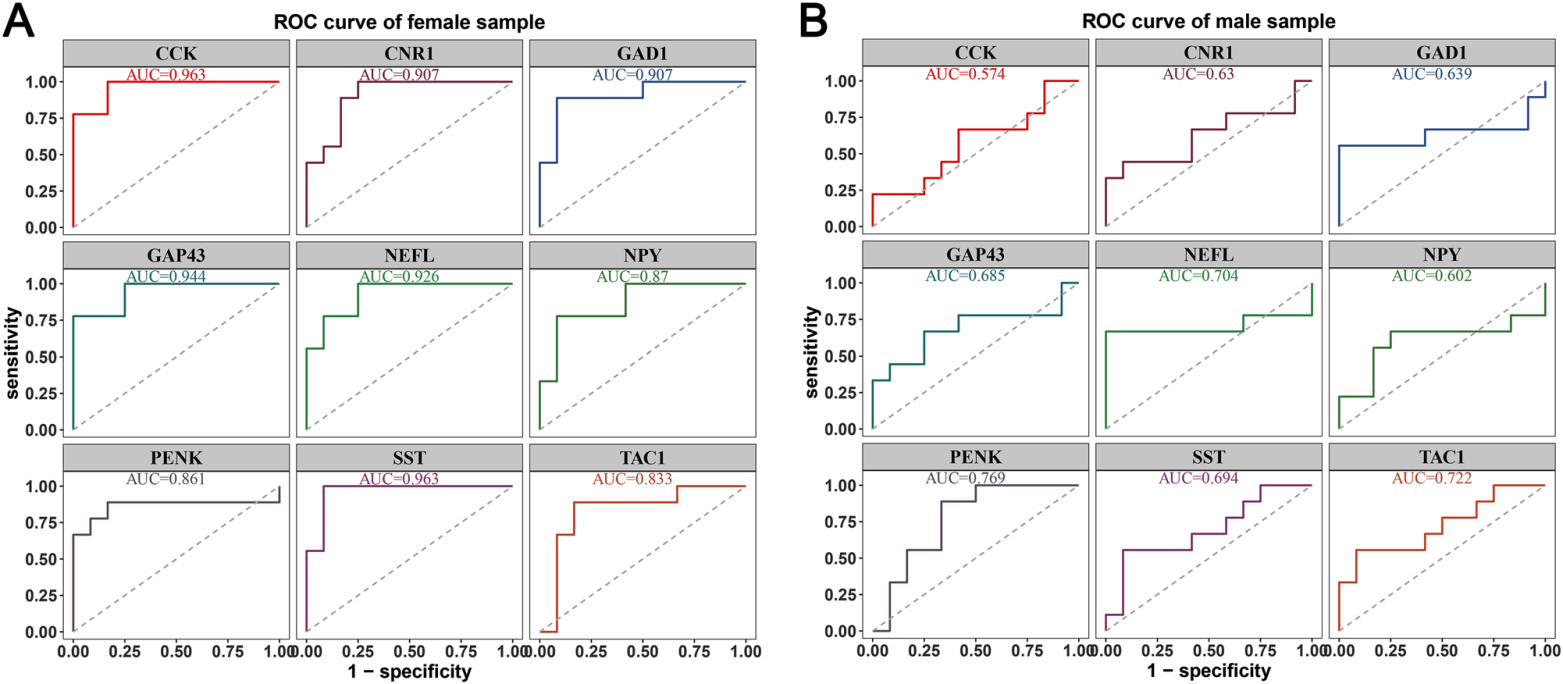
Gender-specific effect of 9 hub genes. The AUC values of 9 hub genes were greater than 0.7. The results demonstrated that 9 hub genes had high diagnostic accuracy in the female patients with AD. For the male patients, NEFL, PENK, and TAC1 were identified with high diagnostic accuracy with AUC values greater than 0.7.

We further performed ROC curve analysis of the male and female samples in GSE48350 to evaluate the gender-specific effect of the above 9 hub genes. As illustrated in Fig. 7, the AUC values of CCK, CNR1, GAD1, GAP43, NEFL, NPY, PENK, SST, and TAC1 were 0.963, 0.907, 0.907, 0.944, 0.926, 0.87, 0.861, 0.963 and 0.833, respectively (Fig. 7A). The results demonstrated that the 9 hub genes had high diagnostic accuracy in the female patients with AD. For the male samples in GSE48350, NEFL, PENK, and TAC1 were identified with high diagnostic accuracy and the AUC values were 0.704, 0.769, and 0.722, respectively (Fig. 7B).

**Figure 8:**
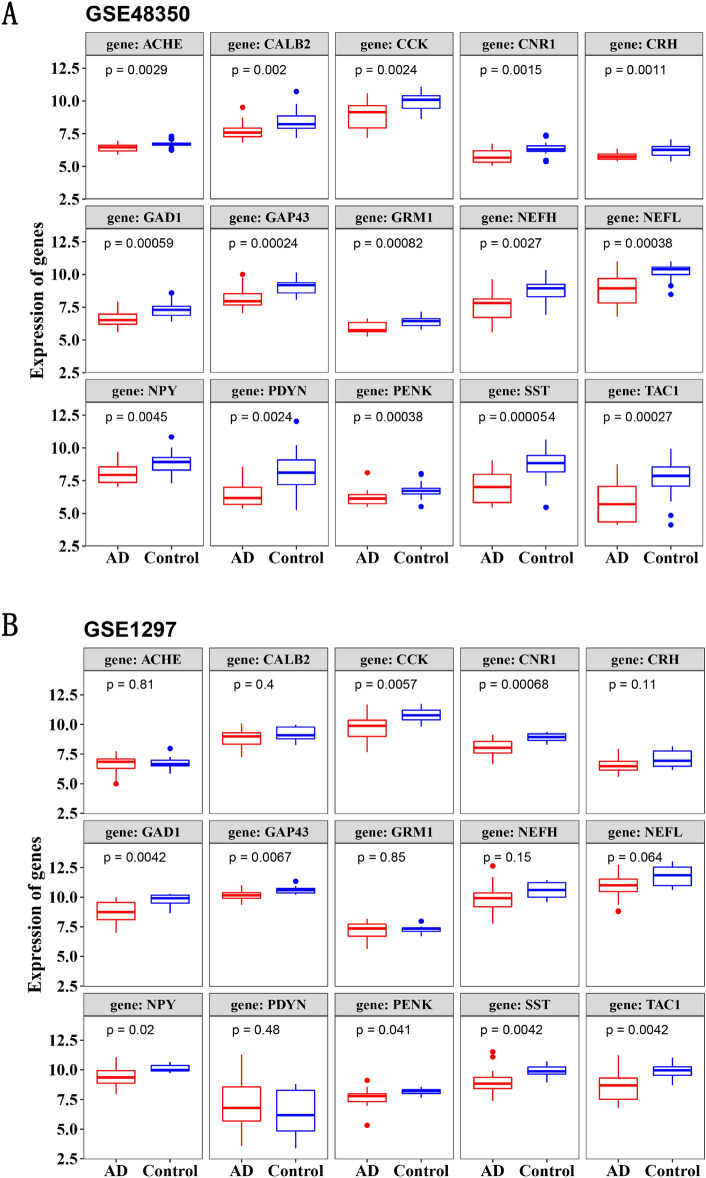
Comparison of diagnostic genes expression between AD and control samples in training dataset GSE48350 (**A**) and external validation dataset GSE1297 (**B**). The 9 diagnostic genes including CCK, CNR1, GAD1, GAP43, NEFL, NPY, PENK, SST, and TAC1 were significantly reduced in AD samples compared with the control group.

The gene expression levels of 9 diagnostic genes were significantly reduced in AD samples compared to control samples in GSE48350 and GSE1297 (Fig. 8A,B).

### Verification of diagnostic genes in the clinical samples

**Figure 9:**
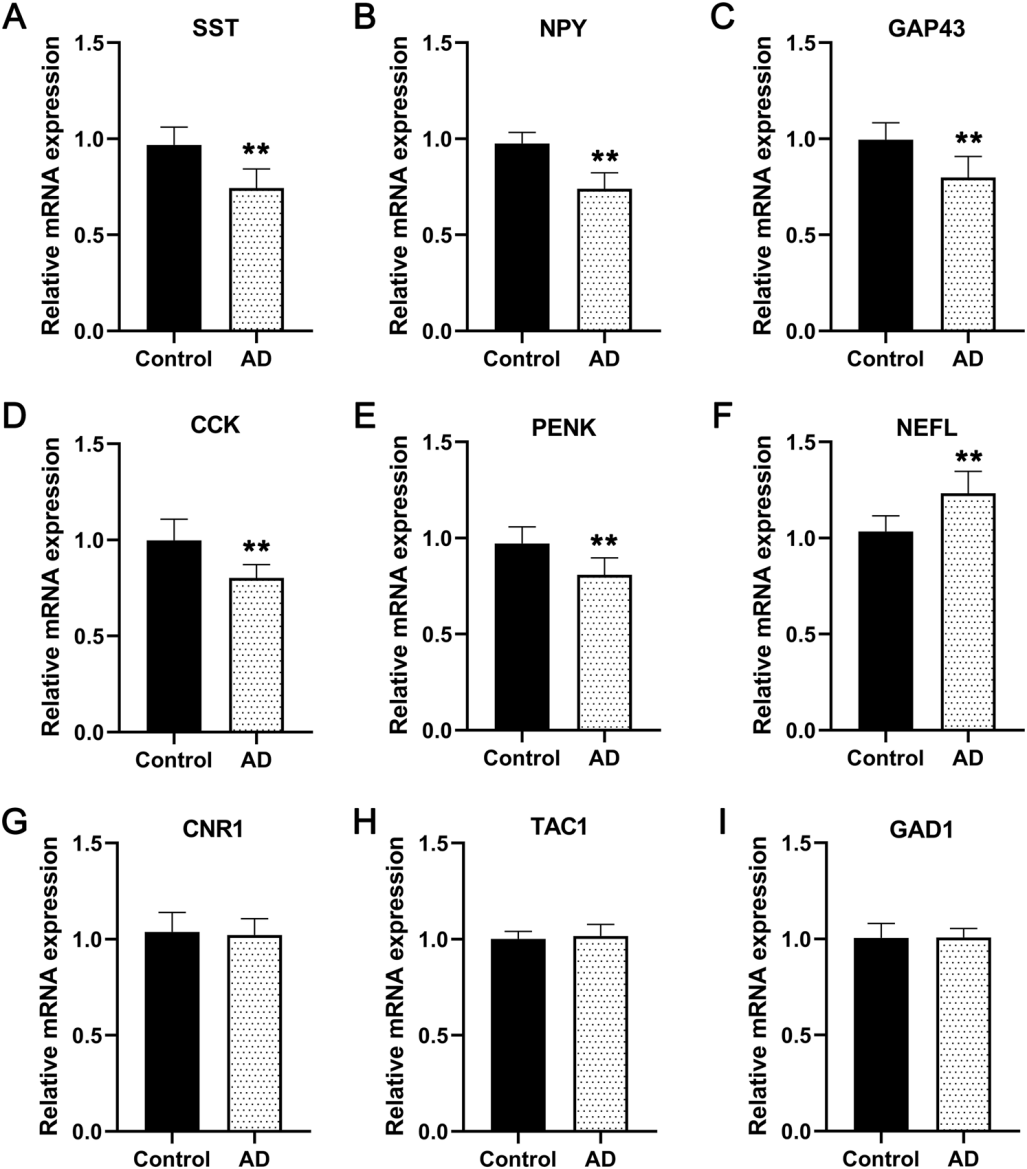
Experimental validation of diagnostic genes by qRT-PCR analysis. Data revealed the downregulation of SST, NPY, GAP43, CCK, and PENK and upregulation of NEFL in the blood of AD patients compared to healthy control subjects. *P-value < 0.05 and **P-value < 0.01.

We next performed qRT-PCR experiments to validate the expression of diagnostic genes in the blood samples from AD patients. The data showed that the mRNA expression level of SST, NPY, GAP43, CCK, and PENK in AD significantly decreased compared with that of the control (Fig. 9; all P < 0.01). Conversely, the opposite result was observed for NEFL (P < 0.01). There were no significant differences between the two groups in terms of CNR1, TAC1, and GAD1 expression.

### Functional enrichment analysis of diagnostic genes

**Figure 10:**
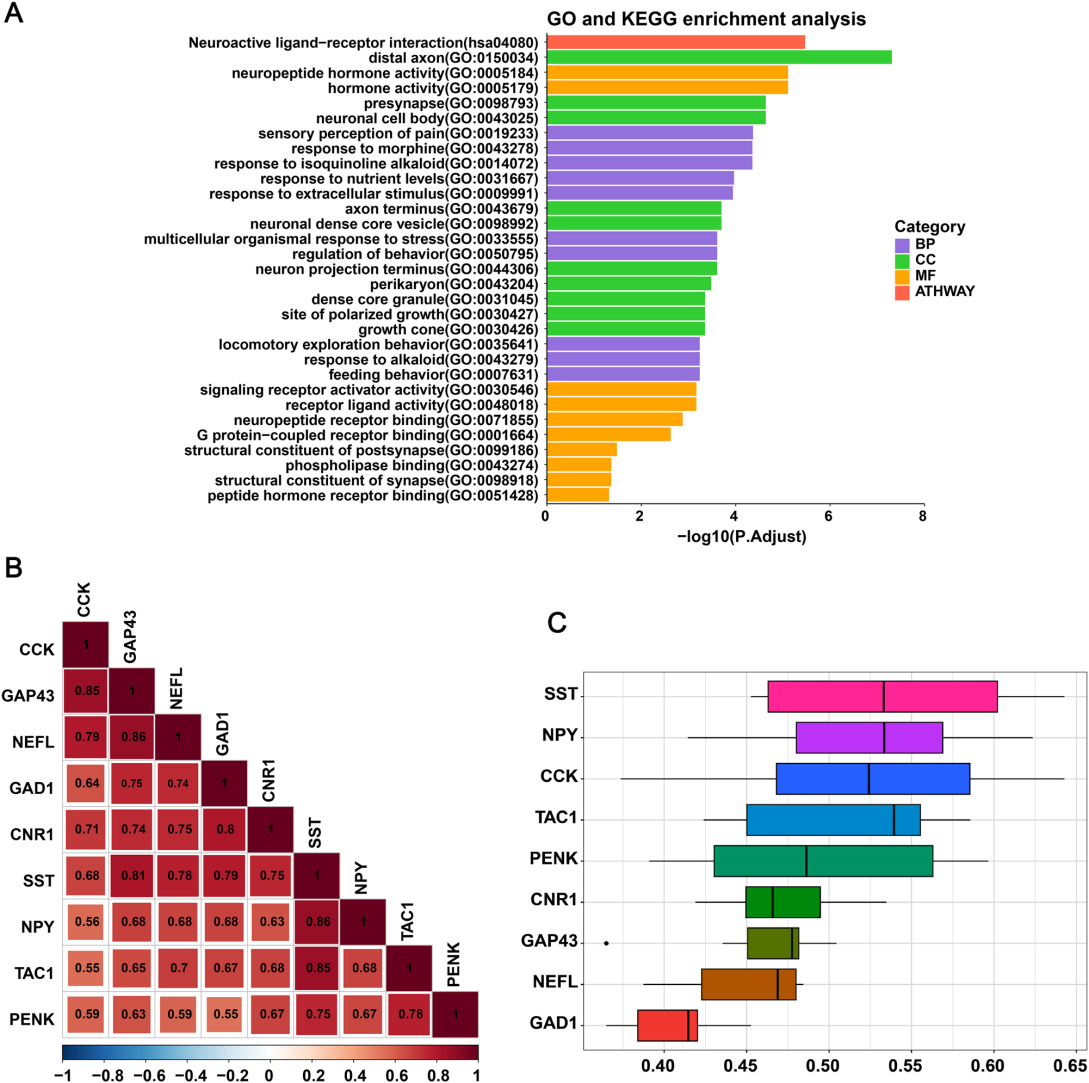
(**A**) Functional enrichment analysis of 9 diagnostic genes. The GO and KEGG enrichment analysis indicated that 9 diagnostic genes were mainly enriched in distal axon, sensory perception of pain, neuropeptide hormone activity, and neuroactive ligand-receptor interaction. (**B**) Correlations analysis of diagnostic genes. There was a positive correlation between genes. Among them, NEFL and GAP43, SST and NPY had the strongest correlation. (**C**) Functional similarity analysis of 9 diagnostic genes. SST had the highest functional similarity score.

The GO and KEGG enrichment analysis indicated that 9 diagnostic genes were mainly enriched in pathways related to distal axon, sensory perception of pain, neuropeptide hormone activity, and neuroactive ligand-receptor interaction (Fig. 10A).

### Correlation analysis and functional similarity analysis of diagnostic genes

The correlations among 9 diagnostic genes were analyzed in GSE48350 (Fig. 10B). There was a significant positive correlation among hub genes. Of these, NEFL and GAP43, as well as SST and NPY, had the strongest correlation with high correlation coefficients at 0.86. The results of functional similarity revealed a higher functional similarity of four hub genes including SST, NPY, CCK, and TAC1 (similarity score > 0.5) (Fig. 10C). Among them, SST had the highest functional similarity score.

### Correlation analysis between diagnostic genes and immune cells

**Figure 11:**
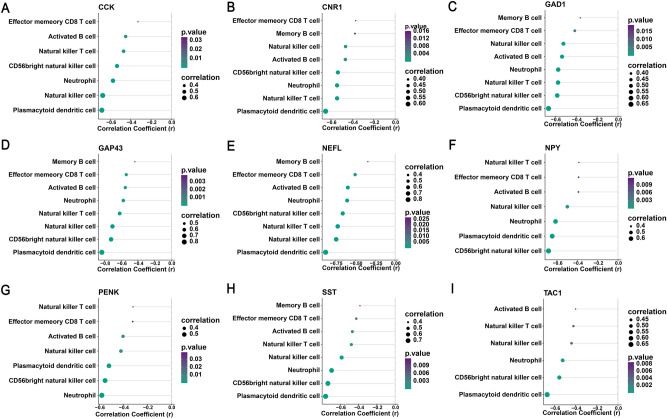
Correlation analysis between diagnostic genes and immune cells. Five hub genes including CNR1, GAD1, GAP43, NEFL, and SST, had significantly negative relationship with the differentially infiltrating immune cells including effector memeory CD8 T cell, activated B cell, memory B cell, natural killer cell, CD56 bright natural killer cell, natural killer T cell, plasmacytoid dendritic cell, and neutrophil.

For the purpose of better understanding the role of 9 diagnostic genes in immune infiltration, we performed spearman correlation analysis to determine the correlation of hub genes with immune cell infiltration. Correlation analysis showed significantly negative correlation of 5 hub genes (CNR1, GAD1, GAP43, NEFL, and SST) with the differentially infiltrating immune cells (effector memory CD8 T cell, activated B cell, memory B cell, natural killer cell, CD56 bright natural killer cell, natural killer T cell, plasmacytoid dendritic cell, and neutrophil) (P < 0.05) (Fig. 11). Other hub genes (CCK, NPY, TAC1, and PENK) also showed negative correlation with partial differentially infiltrating immune cells. Plasmacytoid dendritic cell (pDC) showed the strongest correlation with all hub genes compared with other differentially infiltrating immune cells.

### Identification of potential therapeutic agents of diagnostic genes

**Figure 12:**
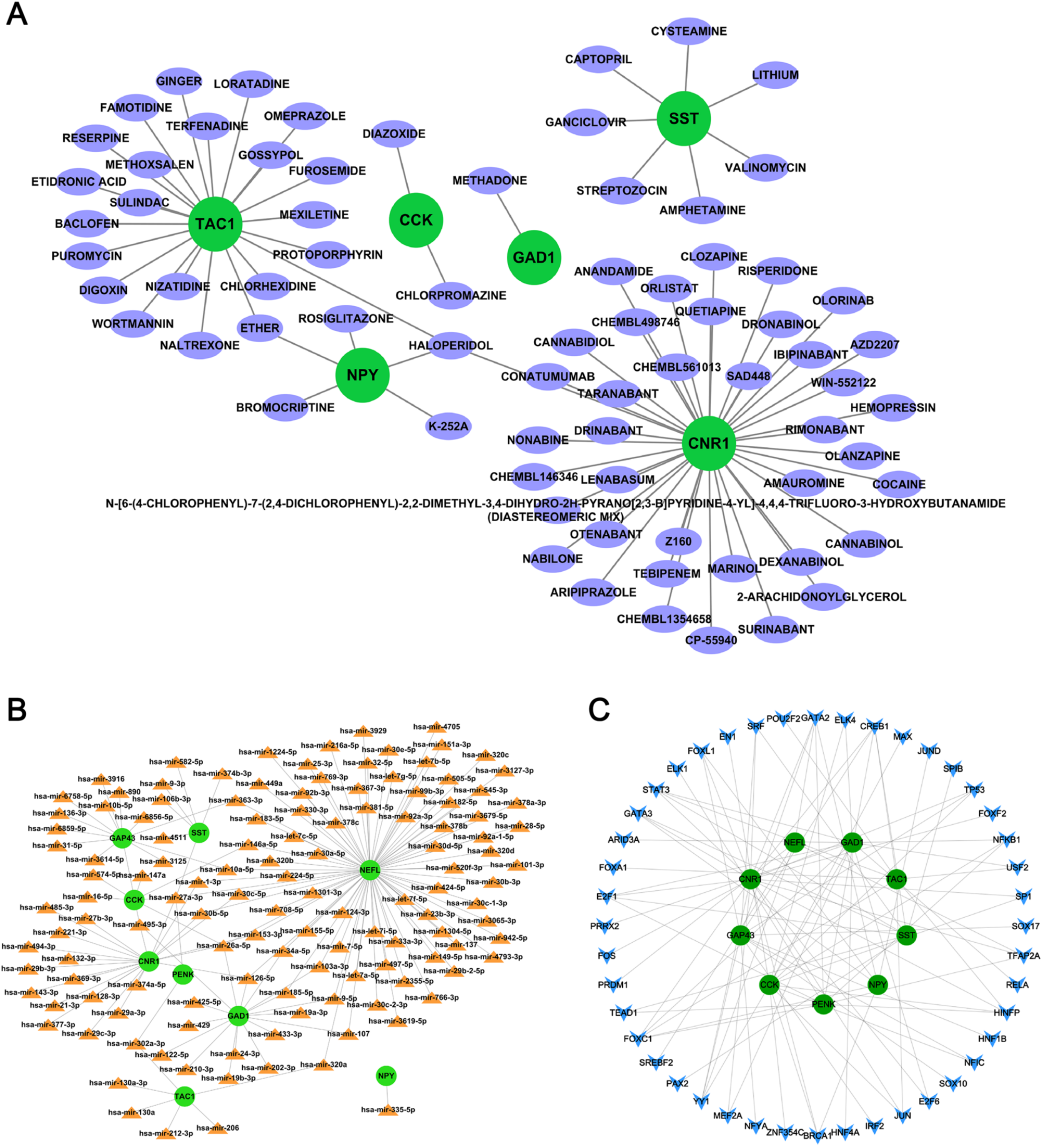
(**A**) Drug–gene interaction diagram. Green circle indicated the related diagnostic gene and pourpre ellipse indicated the drug. A total of 76 candidate drugs for AD treatment were identified by using DGIdb database. (**B**) Potential miRNAs regulatory networks. The interaction network consisted of 9 diagnostic genes and 152 miRNAs. The top three hub genes were NEFL (modulated by 79 miRNAs), CNR1 (modulated by 22 miRNAs), and GAD1 (modulated by 17 miRNAs). (**C**) Potential TFs regulatory networks. The interaction network consisted of 9 diagnostic genes and 91 TFs. The top three hub genes were CNR1 (modulated by 19 TFs), GAD1 (modulated by 13 TFs), and GAP43 (modulated by 12 TFs). GATA2, FOXC1, and CREB1 shared the closest interactions with the diagnostic genes.

We tried to identify potential therapeutic agents modulating the expression of diagnostic genes in AD by using DGIdb. A total of 76 candidate agents for AD treatment were identified (Fig. 12A and Supplementary Table 1). In this study, we identified 39 agents (i.e. nabilone and olorinab) targeting CNR1 expression, 22 agents (i.e. digoxin and haloperidol) targeting TAC1 expression, 7 agents (i.e. cysteamine and streptozocin) targeting SST expression, 5 agents (i.e. rosiglitazone and bromocriptine) targeting NPY expression, 2 agents (chlorpromazine and diazoxide) targeting CCK expression, and 1 agent (methadone) targeting GAD1 expression. There was no specific type of interaction between 20 candidate agents and hub genes. Further investigation of these agents was still required. Specific types of interactions between other 56 candidate agents with hub genes were reported and visualized using Cytoscape software. Among the screened agents, 32 agents were reported potentially related to treatment of AD. Additionally, we did not identify any agents targeting NEFL, GAP43, or PENK in this database.

### Prediction of potential miRNAs regulatory networks of diagnostic genes

As illustrated in Fig. 12B, the interaction network consisted of 9 diagnostic genes and 152 miRNAs. NEFL was modulated by 79 miRNAs (i.e. hsa-mir-103a-3p and hsa-mir-107). CNR1 was modulated by 22 miRNAs (i.e. hsa-mir-21-3p and hsa-mir-1-3p). GAD1 was modulated by 17 miRNAs (i.e. hsa-mir-122-5p and hsa-mir-202-3p). 13 miRNAs (i.e. hsa-mir-890 and hsa-mir-3125) were found targeting GAP43. 6 miRNAs (i.e. hsa-mir-574-5p and hsa-mir-146a-5p) were found targeting CCK. 6 miRNAs (i.e. hsa-mir-106b-3p and hsa-mir-374b-3p) were found targeting SST, and 6 miRNAs (i.e. hsa-mir-130a-3p and hsa-mir-206) were found targeting TAC1. 2 miRNAs (i.e. hsa-mir-27a-3p and hsa-mir-302a-3p) were found targeting PENK, and hsa-mir-335-5p was found targeting NPY. In this network, hsa-mir-27a-3p regulated the largest number of hub genes with the highest connectively degree (= 5).

### Prediction of potential TFs regulatory networks of diagnostic genes

The interaction network consisted of 9 diagnostic genes and 91 TFs (Fig. 12C). CNR1 was modulated by 19 TFs (i.e. BRCA1 and FOS). GAD1 was modulated by 13 TFs (i.e. ARID3A and PRRX2), and GAP43 was modulated by 12 TFs (i.e. CREB1 and GATA2). 11 TFs (i.e. ZNF354C and BRCA1) were found targeting CCK, and 9 TFs (i.e. CREB1 and FOXC1) were found targeting PENK. 8 TFs (i.e. SPIB and CREB1) were found targeting SST, and 8 TFs (i.e. JUN and CREB1) were found targeting TAC1. 6 TFs (i.e. BRCA1 and JUND) were found targeting NPY, and 5 TFs (i.e. GATA2 and YY1) were found targeting NEFL. In the gene-TF network, GATA2, FOXC1, and CREB1 showed the close interaction with hub genes.

## Discussion

Available evidences suggest that AD pathogenesis is strongly associated with oxidative stress and immune microenvironment. However, the mechanism of oxidative stress and neuroinflammation contributing to AD pathogenesis is still not well defined. Intensive investigation of the underlying mechanism of oxidative stress and neuroinflammation in AD is prerequisite for developing effective interventions of AD. Via the PPI network and plotting ROC curve analysis, we identified 9 oxidative stress and immune-related hub genes (SST, NPY, GAP43, CCK, PENK, NEFL, CNR1, GAD1, and TAC1) with good diagnostic values in the training dataset GSE48350 and external validation dataset GSE1297. In this study, we uncovered a significant negative correlation between 9 diagnostic genes and 8 differentially infiltrated immune cells. Somatostatin (SST), a well-known neuropeptide, is expressed throughout the brain in two different isoforms, SST-14 and SST-28. SST positive inhibitory neurons exert dendritic inhibition to regulate the firing activity of cortical neurons and maintain excitatory-inhibitory signal balance^31^. SST expression is found significantly reduced in the hippocampus of AD patients^32^. SST has been reported to be involved in regulating brain Aβ peptide metabolism and promoting aggregation of Aβ peptides^32,33^. Of note, our finding showed the highest functional similarity score of SST in functional similarity analysis of 9 diagnostic genes. Neuropeptide Y (NPY) is widely distributed in the nervous system, especially in GABAergic interneurons. Current finding suggests a remarkable impact of NPY in AD by reducing excitotoxicity of glutamate and overactivity of glutamate receptor, decreasing neuroinflammation, preventing of oxidative stress and protecting of hippocampal and cortical cells from necrosis or apoptosis^34^. In this study, we found a close correlation between SST and NPY with high correlation coefficient of 0.86. Growth associated protein 43 (GAP43), an axonal membrane protein and a biomarker of synaptic dysfunction, is essential to neural growth, axonal regeneration, and stabilization of synaptic function. GAP43 expression is downregulated within the brain tissues of AD patients^35^. The decreased level of exosomal GAP43 in blood is recognized as a potential biomarker for prediction of AD at the asymptomatic stage^36^. Neurofilament light chain (NEFL) is the most abundant cytoskeletal protein in large myelinated axons in adult central nervous system. Similar to plasma p-tau181, NEFL has been reported to be independently associated with cognition and neurodegeneration in AD^37^. Plasma NEFL may be superior to plasma t-tau in diagnostic and prognostic performance of AD^38^. Our finding revealed a close correlation between NEFL and GAP43 with relatively high correlation coefficient of 0.86. Neuropeptide cholecystokinin (CCK) highly expresses in brain regions such as cerebral cortex and hippocampus, and selectively binds to CCK-B receptors in brain. CCK is important for memory retention and consolidation. CCK level is proposed as a possible compensatory protection in response to AD pathological progresses especially tau deposition^39^. Cannabinoids, a component of the endocannabinoid system, can temper the proinflammatory response mediated by microglia in chronic neuroinflammation^40^. The expression of cannabinoid receptor 1 (CNR1) in the brain of AD patients is inconsistent^41^. Tachykinin precursor 1 (TAC1) is involved in encoding multiple types of neuropeptides in central nervous system, such as substance P and neurokinin^42,43^. Downregulation of substance P is found in the hippocampus of AD patients^44^. Substance P may play an important role in the processing of non-amyloidogenic amyloid precursor protein^45^. Expression alteration of glutamate decarboxylase 1 (GAD1), a primary GABA synthesizing enzyme, is found in the prefrontal and temporal cortex of AD patients^46^. The specific role of GAD1 in AD is poorly understood. Proenkephalin (PENK), a small endogenous opioid peptide, exerts depressive effects on cardiac and renal function^47^. Expression of midregional PENK A is downregulated in dementia disorders and acute neuroinflammation^48^. Given limited preliminary data, the molecular mechanism of these 9 oxidative stress and immune-related hub genes contributing to AD pathogenesis is still poorly understood. More targeted researches are expected to unveil their roles and values in AD.

The GO and KEGG enrichment analysis indicated that 9 hub genes were mainly enriched in distal axon, neuropeptide hormone activity, neuroactive ligand-receptor interaction. The neuroactive ligand-receptor interaction signaling pathway plays an important role in the regulation of neuron function through modulating transcription factors and gene expression^49^. Disruption of the genes involved in neuroactive ligand-receptor interaction can lead to diminished memory function^50^. Our finding revealed a significant negative correlation between diagnostic genes and differential immune cells. Notably, pDC showed the strongest correlation with all diagnostic genes. pDC is first recognized as an important regulator of the immune response to virus infection because of its capacity of producing large amounts of IFN-α^51^. In recent years, it becomes apparent that pDC performs a broad range of functions, including innate, adaptive, activating, regulative, protective, and pathogenic functions^52^. A recent study reports that dendritic cell (DC)-based vaccination may be useful in the treatment of neurodegenerative diseases^53^. Enhancement of immune response with DC-based vaccine therapy could potentially enhance antibody production, renewal of neuronal cell, and protection of neuronal cell.

To our best knowledge, there is no definitive drug available in AD treatment. Development of effective pharmacological intervention is still a long-standing challenge. We applied the DGIdb database to identify potential therapeutic agents targeting 9 hub genes. A list of 76 potential therapeutic agents against AD were screened. Except for NEFL, GAP43, and PENK, one or more potential therapeutic agents were identified for other hub genes. Among the screened agents, 31 agents have been reported in AD treatment through different mechanisms. For example, cannabidiol can exert a neuroprotective effect and the mechanism may involve the upregulation of pro-caspase 3 expression and simultaneously downregulation of caspase 3 expression^54^. Quetiapine can alleviate psychotic symptoms and hostility of AD subjects via potentiating the anti-butyrylcholinesterase (BuChE) activity of donepezil^55^. Digoxin shows its anti-inflammatory and anti-oxidative effects in animal study on dementia, and significantly reduces memory loss by decreasing hippocampal cell death^56^. Further animal and clinical researches are needed to verify the safety and effectiveness of these candidate agents in AD treatment.

Our study further revealed the features of potential miRNA-hub gene and TF-hub gene regulatory networks, which may provide valuable knowledge about cellular functions and biological processes in AD. Among the 152 miRNAs, hsa-mir-27a-3p appeared to be the most closely related to the 5 hub genes including CNR1, SST, PENK, CCK, and NEFL. Few data are available on the role of hsa-mir-27a-3p in AD. Preliminary evidence shows that hsa-miR-27a-3p expression is downregulated in cerebrospinal fluid of AD patients, which is accompanied by high level of tau protein and low level of β-amyloid protein^57^. Cellular and animal experiments reveal that downregulation of mir-27a-3p expression is critical for lncRNA NEAT1 regulation in AD development^58^. TF-hub gene network analysis suggested that GATA2, FOXC1, and CREB1 shared the closest interactions with the hub genes. GATA-binding protein 2 (GATA2) has been extensively studied in haematologica^59,60^, and recently recognized as a crucial TF in modulating the expression of monoamine oxidase A in neuronal/cardiovascular disease^61^. As a member of the forkhead box transcription factor family, forkhead box C1 (FOXC1) is involved in the development of embryo, multiple organs, and tumor^62^. FOXC1 can positively regulate cell viability and resistance to oxidative stress in eye^63^. FOXC1 is crucial for cerebellar development and FOXC1 loss correlates with the pathogenesis of Dandy-Walker malformation^64^. Cyclic adenosine monophosphate responsive element-binding protein 1 (CREB1), a leucine-zipper TF, is critical for the formation and consolidation of memory^65^. Impairment of CREB signaling can exacerbate cognitive decline in vascular dementia^66^. CREB-phosphorylation in microglia is involved in Aβ-induced neuronal toxicity and transient memory loss^67^. Further experiments are required to fully elucidate the specific roles of hsa-miR-27a-3p, GATA2, FOXC1, and CREB1 in the regulation of oxidative stress and inflammatory immune response in AD pathogenesis.

There were some limitations in present study. Our findings were based on limited genetic data from GEO database. The qRT-PCR analysis was based on a relatively small sample size. Moreover, the key DEGs and pathways related to oxidative stress and inflammatory immune response in AD pathogenesis were not identified according to different stages of AD. The findings presented in this study prompt us to believe that these identified potential biomarkers are specific for AD to some extent. However, whether these biomarkers are specific enough to other types of dementia, still requires further verification.

## Conclusion

Based on overlapping DEGs between oxidative stress and inflammatory immune response in AD pathogenesis, we identified 9 hub genes (SST, NPY, GAP43, CCK, PENK, NEFL, CNR1, GAD1, and TAC1) with good diagnostic values for AD. Furthermore, we revealed the miRNAs and TFs regulatory networks, as well as the potential therapeutic agents targeting these hub genes. Our findings highlighted the importance of genetic factors in oxidative stress and inflammatory immune response and provided new insights for future studies on the molecular mechanisms and therapeutic targets of AD. Further researches are needed to elucidate the clinical application value of biomarkers in AD, and to determine the generalizability of our findings.

## Supplementary Information

Supplementary Table S1.

## Data Availability

All the data we used in our study are publicly accessible at NCBI GEO database (accession number: GSE48350 and GSE1297; https://www.ncbi.nlm.nih.gov/geo/).

## Abbreviations

AD: Alzheimer’s disease
DEGs: Differentially expressed genes
DEOSGs: Differentially expressed oxidative stress genes
WGCNA: Weighted gene co-expression network analysis
PPI: Protein–protein interaction
TFs: Transcription factors
Aβ: Amyloid β-peptide
OSGs: Oxidative stress-related genes
GO: Gene ontology
KEGG: Kyoto encyclopedia of genes and genomes
GSVA: Gene set variation analysis
ROC: Receiver operating characteristic
AUC: Area under the curve
qRT-PCR: Quantitative real-time PCR
DGIdb: Drug gene interaction database
SST: Somatostatin
NPY: Neuropeptide Y
GAP43: Growth associated protein 43
NEFL: Neurofilament light chain
CCK: Cholecystokinin
CNR1: Cannabinoid receptor 1
TAC1: Tachykinin precursor 1
GAD1: Glutamate decarboxylase 1
PENK: Proenkephalin
pDC: Plasmacytoid dendritic cell
DC: Dendritic cell
GATA2: GATA-binding protein 2
FOXC1: Forkhead box C1
CREB1: Cyclic adenosine monophosphate responsive element-binding protein 1
