# Supplementary material for: Identification and validation of oxidative stress and immune-related hub genes in Alzheimer’s disease through bioinformatics analysis
Source: Sci Rep. 2023 Jan 12;13:657. doi: 10.1038/s41598-023-27977-7 (PMC9837191; doi:10.1038/s41598-023-27977-7)
Supplement: Supplementary file 1 — Supplementary Table S1. [file 41598_2023_27977_MOESM1_ESM.docx]

| **Supplementary Table 1** Candidate drugs targeted by the hub genes | | | | | | |
| --- | --- | --- | --- | --- | --- | --- |
| No. | Gene | Drug | Interaction  types | Sources | PMIDs | Reported for AD Treatment |
| 1 | CCK | CHLORPROMAZINE | – | NCI | 3628280 | – |
| 2 | CCK | **DIAZOXIDE** | – | NCI | 1032363 | 20847430 |
| 3 | CNR1 | **NABILONE** | agonist; | TdgClinicalTrial; TTD; | 11698062; 8981483; | 31182351 |
|  |  |  | partial agonist | GuideToPharmacology; TEND | 10575283; 11752352; |  |
|  |  |  |  | ChemblInteractions | 8864542; 17139494 |  |
| 4 | CNR1 | OLORINAB | agonist | GuideToPharmacology | – | – |
| 5 | CNR1 | DRINABANT | antagonist | TdgClinicalTrial; | – | – |
|  |  |  |  | ChemblInteractions |  |  |
| 6 | CNR1 | OTENABANT | antagonist | ChemblInteractions; | – | – |
|  |  |  |  | GuideToPharmacology |  |  |
| 7 | CNR1 | SURINABANT | antagonist | ChemblInteractions; TTD; | – | – |
|  |  |  |  | GuideToPharmacology; |  |  |
|  |  |  |  | TdgClinicalTrial |  |  |
| 8 | CNR1 | **CANNABIDIOL** | allosteric modulator; | ChemblInteractions; | 26218440 | 32109623 |
|  |  |  | modulator; antagonist | GuideToPharmacology |  |  |
| 9 | CNR1 | TARANABANT | antagonist; | ChemblInteractions; | 20423086 | – |
|  |  |  | inverse agonist | DTC; TdgClinicalTrial |  |  |
|  |  |  |  | GuideToPharmacology |  |  |
| 10 | CNR1 | IBIPINABANT | antagonist | ChemblInteractions; | – | – |
|  |  |  |  | GuideToPharmacology |  |  |
| 11 | CNR1 | NONABINE | agonist | ChemblInteractions | – | – |
| 12 | CNR1 | CHEMBL1354658 | agonist | GuideToPharmacology | – | – |
| 13 | CNR1 | **DRONABINOL** | agonist | TdgClinicalTrial; NCI; | 12686391; 11204352; 11752352; | 30374797 |
|  |  |  |  | ChemblInteractions | 17139284; 17016423; 15894066 |  |
| 14 | CNR1 | AZD2207 | antagonist | ChemblInteractions | – | – |
| 15 | CNR1 | RIMONABANT | antagonist | DTC; TdgClinicalTrial; TEND | 23357307; 8636122; 18712856; | – |
|  |  |  |  | GuideToPharmacology; | 19530697; 11752352; 18585913 |  |
| 16 | CNR1 | CHEMBL561013 | agonist | GuideToPharmacology | – | – |
| 17 | CNR1 | SAD448 | agonist | ChemblInteractions | – | – |
| 18 | CNR1 | **ANANDAMIDE** | agonist | GuideToPharmacology | – | 30333717 |
| 19 | CNR1 | LENABASUM | agonist | GuideToPharmacology | – | – |
| 20 | CNR1 | **DEXANABINOL** | agonist | GuideToPharmacology | – | 15728830; 11249704 |
| 21 | CNR1 | CANNABINOL | agonist | GuideToPharmacology | – | – |
| 22 | CNR1 | **HALOPERIDOL** | – | PharmGKB | 23799528; 20107430; 20631561 | 16316485; 29067299 |
| 23 | CNR1 | OLANZAPINE | – | PharmGKB | 23799528; 20107430; 20631561 | – |
| 24 | CNR1 | **CP-55940** | – | DTC | 17027269 | 33252082 |
| 25 | CNR1 | **QUETIAPINE** | – | PharmGKB | 23799528; 20107430; 20631561 | 35083020; 33235619 |
| 26 | CNR1 | **RISPERIDONE** | – | PharmGKB | 23799528; 20107430; 20631561 | 33176899; 28269767 |
| 27 | CNR1 | COCAINE | – | PharmGKB | – | – |
| 28 | CNR1 | CHEMBL146346 | – | DTC | 17110113 | – |
| 29 | CNR1 | CONATUMUMAB | – | TTD | – | – |
| 30 | CNR1 | **HEMOPRESSIN** | – | DTC | 18077343 | 32241604 |
| 31 | CNR1 | Z160 | – | DTC | 19815411 | – |
| 32 | CNR1 | WIN-552122 | – | DTC | 18579386; 17027269 | – |
| 33 | CNR1 | **ARIPIPRAZOLE** | – | PharmGKB | 23799528; 20107430; 20631561 | 34502282; 23350964 |
| 34 | CNR1 | CHEMBL498746 | – | DTC | 19530697 | – |
| 35 | CNR1 | AMAUROMINE | – | DTC | – | – |
| 36 | CNR1 | **ORLISTAT** | – | TTD | – | 19505770 |
| 37 | CNR1 | MARINOL | – | TdgClinicalTrial; TEND; TTD | – | – |
| 38 | CNR1 | DIASTEREOMERIC MIX | – | DTC | 20423086 | – |
| 39 | CNR1 | **2-ARACHIDONOYLGLYCEROL** | – | DTC | 17110113 | 33939165 |
| 40 | CNR1 | TEBIPENEM | – | TTD | – | – |
| 41 | CNR1 | **CLOZAPINE** | – | PharmGKB | 23799528; 20107430; 20631561 | 26742522 |
| 42 | GAD1 | METHADONE | – | PharmGKB | 31866536 | – |
| 43 | NPY | **ROSIGLITAZONE** | – | NCI | 9421288 | 22028424; 33814447 |
| 44 | NPY | **BROMOCRIPTINE** | – | NCI | 1679226 | 34193504 |
| 45 | NPY | **HALOPERIDOL** | – | NCI | 16154634 | 17388716; 29067299 |
| 46 | NPY | ETHER | – | NCI | 9696062 | – |
| 47 | NPY | K-252A | – | NCI | 2259391 | – |
| 48 | SST | CYSTEAMINE | binder | TTD | 2653642; 2901134; 4080089; | – |
|  |  |  |  |  | 11752352; 6142843 |  |
| 49 | SST | **STREPTOZOCIN** | – | NCI | 7664670 | 34323698 |
| 50 | SST | **VALINOMYCIN** | – | NCI | 6129560 | 32905804 |
| 51 | SST | AMPHETAMINE | – | NCI | 2874568 | – |
| 52 | SST | **CAPTOPRIL** | – | NCI | 2864479 | 33060184; 35243060 |
| 53 | SST | GANCICLOVIR | – | NCI | 10766345 | – |
| 54 | SST | **LITHIUM** | – | NCI | 2888503 | 29859917; 34119602 |
| 55 | TAC1 | ETIDRONIC ACID | – | NCI | 16133581 | – |
| 56 | TAC1 | **DIGOXIN** | – | NCI | 1717629 | 35064518; 31269278 |
| 57 | TAC1 | HALOPERIDOL | – | NCI | 6166743 | 17388716; 16316485 |
| 58 | TAC1 | OMEPRAZOLE | – | NCI | 15684503; 16259735 | – |
| 59 | TAC1 | METHOXSALEN | – | NCI | 9430496 | – |
| 60 | TAC1 | **NIZATIDINE** | – | NCI | 12495560 | 11882746 |
| 61 | TAC1 | **RESERPINE** | – | NCI | 6174352; 6183051 | 32244635; 19264117 |
| 62 | TAC1 | PUROMYCIN | – | NCI | 14623771 | – |
| 63 | TAC1 | MEXILETINE | – | NCI | 7686682 | – |
| 64 | TAC1 | **BACLOFEN** | – | NCI | 2467977 | 29133125; 2285270 |
| 65 | TAC1 | GOSSYPOL | – | NCI | 2887120 | – |
| 66 | TAC1 | **SULINDAC** | – | NCI | 1707646 | 17266991; 26900939 |
| 67 | TAC1 | CHLORHEXIDINE | – | NCI | 14631229 | – |
| 68 | TAC1 | ETHER | – | NCI | 2421032 | – |
| 69 | TAC1 | **FUROSEMIDE** | – | NCI | 2048822 | 33225679; 34118718 |
| 70 | TAC1 | **WORTMANNIN** | – | NCI | 9850167 | 11866882; 10582589 |
| 71 | TAC1 | LORATADINE | – | NCI | 1283698; 9055977 | – |
| 72 | TAC1 | FAMOTIDINE | – | NCI | 12495560 | – |
| 73 | TAC1 | **NALTREXONE** | – | NCI | 7536308 | 3541052; 3903533; |
| 74 | TAC1 | PROTOPORPHYRIN | – | NCI | 9677143 | – |
| 75 | TAC1 | TERFENADINE | – | NCI | 1696438 | – |
| 76 | TAC1 | **GINGER** | – | NCI | 1282221 | 33378982; 34279199 |
|  |  |  |  |  |  |  |

These data were obtained from the DGIdb database; Drugs in bold are reported in AD treatment; No38 represent N-[6-(4-CHLOROPHENYL)-7-(2,4-DICHLOROPHENYL)-2,2-DIMETH-YL-3,4-DIHYDRO-2H-PYRANO[2,3-B]PYRIDINE-4-YL]-4,4,4-TRIFLUORO-3-HYDROXYBUTANAMIDE (DIASTEREOMERIC MIX)
